# Circulating angiopoietin-2 and soluble Tie-2 in type 2 diabetes mellitus: a cross-sectional study

**DOI:** 10.1186/1475-2840-10-55

**Published:** 2011-06-23

**Authors:** Sazan Rasul, Marie Helene Reiter, Aysegul Ilhan, Katharina Lampichler, Ludwig Wagner, Alexandra Kautzky-Willer

**Affiliations:** 1 Department of Internal Medicine III, Division of Endocrinology and Metabolism, Unit of Gender Medicine, Medical University of Vienna, Vienna, Austria; 2 Department of Internal Medicine III, Division of Endocrinology and Metabolism, Medical University of Vienna, Vienna, Austria; 3 Department of Internal Medicine III, Division of Nephrology and Dialysis, Medical University of Vienna, Vienna, Austria

## Abstract

**Background:**

Type 2 diabetes is associated with increased levels of Angiopoietin-2 (Ang-2) and soluble Tie-2 (sTie-2), but its impact on vascular disease is still unknown. This study aimed to further explore the associations of Ang-2 and sTie-2 with metabolic control and diabetic complications.

**Methods:**

In a cross-sectional designed study, levels of Ang-2 and sTie-2 as well as their relationships to cardiometabolic parameters were determined in 80 type 2 diabetic subjects (age 65 ± 7 years, female 47.4%).

**Results:**

After controlling for age and BMI, Ang-2 levels were associated with levels of sTie-2, diastolic blood pressure, plasma insulin, homeostasis model assessment of insulin resistance (HOMA-IR), creatinine, glomerular filtration rate (GFR), and gamma-glutamyl transferase (GGT) (all p < 0.02). Presence of diabetic macrovascular complications, polyneuropathy and insulin therapy were associated with higher Ang-2 levels (p < 0.05). Conversely, sTie-2 levels were associated with glycated hemoglobin (HbA_1c_), fasting plasma glucose and insulin, HOMA-IR, triglyceride, and liver function parameters (all p < 0.03). Multiple linear regression analysis showed that Ang-2 remained significantly associated only with levels of GGT (p < 0.04), whereas sTie-2 remained significantly associated with HbA**_1c_**, insulin levels, and HOMA-IR (p < 0.03). No differences in Ang-2 and sTie-2 levels were observed with regard to gender of participants.

**Conclusions:**

Ang-2 is independently associated with levels of GGT while sTie-2 is independently associated with levels of HbA**_1c_**, plasma insulin and HOMA-IR in type 2 diabetic subjects. Therefore we suggest that the associations of Ang-2 and sTie-2 with type 2 diabetes are based on different patho-physiological mechanisms.

## Background

Angiopoietins are growth factors that promote angiogenesis together with vascular endothelial growth factor (VEGF). Among the four identified angiopoietins (1-4), Angiopoietin (Ang)-1 and Ang-2 are reported to be required for the formation of mature blood vessels, as demonstrated by mouse knock out studies [1,2]. Ang-2 is expressed primarily in the vascular endothelium at sites of vascular remodeling [3]. Both Ang-1 and Ang-2 act by binding to the endothelium-specific receptor tyrosine kinase 2 (Tie-2). A soluble form of the Tie-2 receptor can be detected in human biological fluids [4]. The Ang/Tie system tightly controls the endothelial phenotype during angiogenesis and vascular inflammation in a unique fashion. While Ang-1 has an agonistic effect on Tie-2 through induction of autophosphorylation of the receptor necessary for the stabilization of the blood vessels [5], Ang-2 appears to have an antagonistic effect. It acts as a competitive inhibitor of Ang-1 for Tie-2 binding, thereby inhibiting Ang-1/Tie-2 signaling [6]. Consequently, the loss of Tie-2 signaling destabilizes the endothelium and facilitates the angiogenic and inflammatory response to growth factors and cytokines [7]. Ang-2 promotes also VEGF induced neovascularization.

Growing evidence suggests an involvement of Ang-2 and its receptor Tie-2 in the pathophysiology of different vascular and inflammatory diseases such as arteriosclerosis [8], hypertension [9], idiopathic pulmonary arterial hypertension [10], chronic kidney disease [11], and rheumatoid arthritis [12].

Type 2 diabetes mellitus is a metabolic disease characterized by chronic hyperglycemia which mainly results from a deficiency in peripheral insulin effects (insulin resistance). However, the morbidity and mortality of diabetes are mainly attributed to the development of both macrovascular and microvascular complications. Among factors such as obesity, hypercholesterolemia, hyperlipidemia, increased formation of advanced glycation end-products [13] and increased oxidative stress [14], a dysfunction in angiogenesis also has been suggested as a common origin for diabetic vascular complications [15]. In addition, previous studies reported an elevation of plasma levels of VEGF, Ang-2 and soluble Tie-2 (sTie-2) in subjects with type 2 diabetes mellitus [16,17]. A selective increase of plasma levels of Ang-2 and sTie-2, but not Ang-1, is accompanied by neovascularization and endothelial abnormalities. Endothelial abnormalities are closely linked to the pathophysiology of microvascular and atherosclerotic vascular complications in type 2 diabetes [18,19]. Although a previous study showed that raised levels of plasma Ang-2 and VEGF in diabetes are independent of concomitant vascular disease [20], the underlying mechanisms for the association of Ang-2 and sTie-2 with type 2 diabetes are not well understood. Up to now, there are no published data on plasma levels of both Ang-2 and sTie-2 in subjects with type 2 diabetes mellitus evaluating their relationships with metabolic and glycaemic parameters, liver and renal function, and lipid profile. Therefore, in this cross-sectional study, we aimed to further explore the relationship between circulating Ang-2 and sTie-2 levels in type 2 diabetic subjects and to identify factors that might influence or predict their levels.

## Methods

The study was approved by the Medical University of Vienna Ethics Committee and included 80 type 2 diabetic subjects (age 65 ± 7 years, time since diagnosis of diabetes 15 ± 9 years, BMI 32 ± 6, shown as mean ± standard deviation). All participants were recruited at the Outpatient Clinic of Diabetes, Division of Endocrinology and Metabolism, Department of Internal Medicine III. Written informed consent was provided by all participants prior to the study. The participants fulfilled the following inclusion criteria: established type 2 diabetes mellitus (diagnosed according to WHO criteria [21]), age 40-80 years, under oral anti-diabetic and/or insulin therapy, intact hepatic function as evaluated by aspartate aminotransferase (AST) or alanin aminotransferase (ALT) not more than 2 times the upper reference limit and intact renal function as evaluated by glomerular filtration rate > 50 mL/min/1.73 m^2^, no history of recent (< 6 months) cardiac, cerebral or peripheral infarction, no history of Charcot's disease or chemotherapy, and no glucocorticoid or other hormone substitution therapy. Presence of diabetic polyneuropathy was established by evaluation of nerve conduction velocity for upper and lower extremities. Presence of overt macrovascular complications was established by a history of previous (> 6 months) stroke, myocardial infarction, angina, and coronary or peripheral revascularization.

Prior to blood sampling, a disease-specific questionnaire concerning duration since diagnosis of diabetes, type of anti-diabetic therapy, incidence of diabetic complications, presence of any other chronic disease and cigarette and alcohol consumption of the patients was filled out with the participants. Blood pressure, height, and waist circumference of all participants were measured and body mass index (BMI) was determined. Fasting venous blood samples were obtained from a cubital vein. One sample was allowed to clot for approximately one hour on ice and was then centrifuged at 3000 rpm for 10 minutes. Plasma was separated and stored in aliquots at -28°C until Ang-2 and sTie-2 were analyzed. In a routine biochemistry laboratory, glycated hemoglobin (HbA**_1c_**) was determined (Haemoglobin Testing System, D-10, Bio-Rad Laboratories, Inc) and levels of fasting plasma glucose (using the hexokinase method), C-peptide, insulin, lipid profile, liver and renal function parameters were measured in the central clinical laboratory of the General Hospital of Vienna, Vienna, Austria using commercially available assays. Furthermore, urine samples were obtained from all participants and were tested for the presence of glucose, protein and albumin. In addition, HOMA-IR was calculated by multiplying the value of fasting plasma glucose (mg/dl) by the value of fasting plasma insulin (μU/ml) divided by 405. Waist to height ratio was calculated by dividing the waist circumference by the height (both in cm) of participants. Glomerular filtration rate was estimated using MDRD Study Equation [22]. Circulating levels of plasma Ang-2 and sTie-2 were assayed using commercially available Quantikine human ELISA kits (R&D systems, Abingdon, UK). The intra-and inter-assay coefficients of variation were 6.5% and 9.1%, respectively, for Ang-2 and 4.4% and 6.5%, respectively, for Tie-2. The assays were performed according to the manufacturer's instructions.

### Statistical analysis

Collected data were subjected to the Kolmogorov-Smirnov test to determine their distribution. Not normally distributed data were log (base 10)- transformed for all following analyses and presented as median and range. Statistical comparisons of the obtained data were performed using independent samples t-tests. Pearson's correlations test was used to determine the associations of Ang-2 and Tie-2 with age and BMI. Partial (two-tailed) correlation tests adjusted for age and BMI were used to study the associations of Ang-2 and Tie-2 to other measured variables. Linear multiple regression analysis was used to determine independent predictors for Ang-2 and sTie-2. For this analysis, variables that showed a *p*-value < 0.05 with Ang-2 or sTie-2 in the partial correlation test were included in the analysis as independent variables. For all statistical tests, a value of *p *< 0, 05 was considered to be significant. Data entry and analysis were performed using SPSS version 17.0 (Chicago, Illinois).

## Results

### 1. Clinical characteristics of type 2 diabetic participants and circulating levels of Ang-2 and sTie-2

Eighty diabetic subjects (47.7% female) were studied. Among them, 50% presented with diabetic polyneuropathy (50% female), 29.1% with overt diabetic macrovascular complications (mostly cardiovascular complications with no significant difference in sex distribution and were all documented), 3.8% with microalbuminuria and 3.8% with diabetic retinopathy (documented). In addition, 48.7% were on insulin therapy and 23% were active smokers. Clinical and demographic characteristics of the type 2 diabetic participants and the levels of plasma Ang-2 and sTie-2 are shown in (Table 1). Levels of Ang-2 were significantly positively associated with BMI, whereas levels of sTie-2 were negatively associated with age of participants. Levels of Ang-2 and sTie-2 did not differ significantly between sexes and were independent of smoking status of the participants. No statistically significant associations were observed between levels of Ang-2 or sTie-2 and the waist to height ratio of participants or duration since diagnosis of diabetes.

**Table 1:** Clinical and demographic characteristics of type 2 diabetic participants and their relationships with levels of Ang-2 and Tie-2 after controlling for age and BMI

Parameters	Participants (n = 80)	*p- *value
Sex (%)	47.7 (female)	NS

Age (years)	65 ± 7	**^‡^**(r = -0.349, *p *< 0.002)

BMI (kg/m^2^)	32 ± 6	**^†^**(r = 0.277, *p *< 0.01)

Waist to height ratio	0.66 ± 0.12	NS

Duration of diabetes (years)	15 ± 9	NS

*Ang-2 (pg/ml)	2955 (1349-9000)	**^‡^**(r = 0.355, *p *< 0.002)

sTie-2 (ng/ml)	28.34 ± 7.58	**^†^**(r = 0.355, *p *< 0.002)

Active smokers (%)	23	NS

Therapy		**^†^**(*p *< 0.04)
Insulin therapy (%)	48.7	
Oral anti-diabetic therapy (%)	51.3	

Systolic blood pressure (mmHg)	145 ± 21	NS

Diastolic blood pressure (mmHg)	82 ± 14	**^†^**(r = -0.259, *p *< 0.02)

Diabetic retinopathy (%)	3.8	NS

Microalbuminuria (%)	3.8	NS

Polyneuropathy (%)	50	**^†^**(*p *< 0.01)

**^#^**Macro-vascular complications (%)	29.1	**^†^**(*p *< 0.009)

Data are expressed as mean ± standard deviation or as percentage. (*): data not normally distributed, expressed as a median and range, and log_10- _transformed for analysis. (^#^): included cardiac, cerebral or peripheral vascular disease. (**^†^**): significantly associated with levels of Ang-2. (**^‡^**): significantly associated with levels of sTie-2. NS: not significant.

### 2. Ang-2 relationships in type 2 diabetic subjects

**Figure 1:**
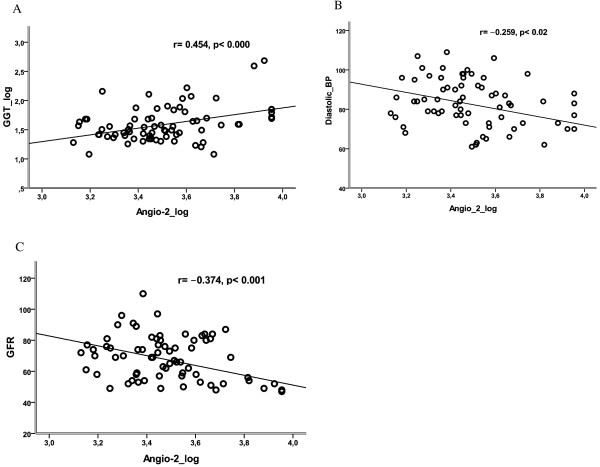
**Associations of Ang-2 with the levels of GGT (A), diastolic blood pressure (B), and GFR (C), in type 2 diabetic subjects**.

After controlling for age and BMI, levels of plasma Ang-2 were positively associated with levels of sTie-2 (r = 0.355, *p *= 0.002), plasma levels of insulin, creatinine, GGT (Figure 1A) and HOMA-IR, whereas they were negatively associated with diastolic blood pressure (Figure 1B) and GFR (*p *= 0.001) (Figure 1C, Table 2). In addition, Ang-2 levels were significantly higher among subjects with insulin therapy, diabetic polyneuropathy, and diabetic macro-angiopathy (Table 1). Importantly, multivariate analysis tests controlled for diabetic therapy, diabetic polyneuropathy, serum creatinine, and levels of GGT, showed that Ang-2 remained significantly higher among diabetic subjects with macrovascular disease when compared to those without macrovascular complications. However, levels of Ang-2 did not remain significantly different between participants with or without diabetic polyneuropathy after controlling for the presence of macrovascular complications. Interestingly, linear multiple regression analysis showed that plasma GGT levels are the only predictor for circulating plasma Ang-2 in type 2 diabetes mellitus (Table 3). Furthermore, no statistically significant associations were observed between plasma levels of Ang-2 and the waist to height ratio, duration since diagnosis of diabetes, and systolic blood pressure. No significant associations were observed between Ang-2 levels and the levels of testosterone in male (mean 3.45 ± 1.5 ng/ml) as well as in female (mean 0.16 ± 0.13 ng/ml) participants. In addition, no significant associations were observed between levels of Ang-2 and the levels of estradiol in female [median 9, range (9-128) pg/ml] and in male [median 24, range (13-70) pg/ml] participants. As expected, because of few reported cases, no significant differences were observed in the levels of circulating Ang-2 in subjects with diabetic retinopathy, or microalbuminuria.

**Table 2:** Laboratory parameters of type 2 diabetic participants and their relationships with levels of Ang-2 and sTie-2 after controlling for age and BMI

Parameters	Participants (n = 80)	*p- *value
*HbA_1c _(%)	7.6 (6-12.9)	**^‡^**(r = 0.526, *p *< 0.000)

Fasting blood Glucose (mg/dl)	152 ± 55	**^‡^**(r = 0.353, *p *< 0.005)

C-Peptide (ng/ml)	2.5 ± 1.3	NS

*Insulin (μU/ml)	11 (1-99.7)	**^†^**(r = 0.355, *p *< 0.004), **^‡^**(r = 0.289, *p *< 0.02)

*HOMA-IR	4 (0-57.7)	**^†^**(r = 0.334, *p *< 0.007), **^‡ ^**(r = 0.322, *p *< 0.01)

Total cholesterol (mg/dl)	187 ± 47	NS

LDL-cholesterol (mg/dl)	102 ± 40	NS

*HDL-cholesterol (mg/dl)	44 (26-117)	NS

Triglyceride (mg/dl)	198 ± 109	**^‡^**(r = 0.305, *p *< 0.01)

Creatinine (mg/dl)	1.09 ± 0.27	**^†^**(r = 0.345, *p *< 0.003)

GFR (ml/min/1.73 m^2^)	66 ± 15	**^†^**(r = -0.374, *p *< 0.001)

*CRP (mg/dl)	0.27 (0.01-3.2)	NS

Bilirubin (mg/dl)	0.62 ± 0.22	NS

*AST (U/l)	24 (12-53)	**^‡^**(r = 0.452, *p *< 0.000)

ALT(U/l)	27 ± 11	**^‡^**(r = 0.369, *p *< 0.002)

*GGT(U/l)	32 (8-165)	**^†^**(r = 0.454, *p *< 0.000), **^‡^**(r = 0.483, *p *< 0.000)

*SHBG (nmol/l)	33 (12.8-189)	NS

Data are expressed as mean ± standard deviation. (*): data not normally distributed, expressed as a median and range, and log_10- _transformed for analysis. HOMA-IR: homeostasis model assessment for insulin resistance, LDL: low density lipoprotein, HDL: high density lipoprotein, GFR: glomerular filtration rate, CRP: C-reactive protein, AST: aspartate aminotransferase. ALT: alanin aminotransferase, GGT: gamma-glutamyl transferase, SHBG: sex hormone binding globulin. (**^†^**): significantly associated with levels of Ang-2. (**^‡^**): significantly associated with levels of sTie-2. NS: not significant.

**Table 3:** Multiple regression analysis with levels of plasma Ang-2 as dependent factor in studied type 2 diabetic subjects

Parameters	Standard error	p-value
BMI	0.004	0.11

Therapy	0.056	0.89

Diastolic blood pressure	0.002	0.09

Polyneuropathy	0.050	0.09

Macrovascular complications	0.058	0.87

*Insulin	0.180	0.56

*HOMA-IR	0.162	0.66

Creatinine	0.140	0.80

GFR	0.002	0.08

*GGT	0.080	**0.04**

Significant *p-values *are highlighted in bold

(*): data not normally distributed and log_10- _transformed for analysis. BMI: body mass index, HOMA-IR: homeostasis model assessment for insulin resistance, GFR: glomerular filtration rate, GGT: gamma-glutamyl transferase.

### 3. sTie-2 relationships in type 2 diabetic subjects

**Figure 2:**
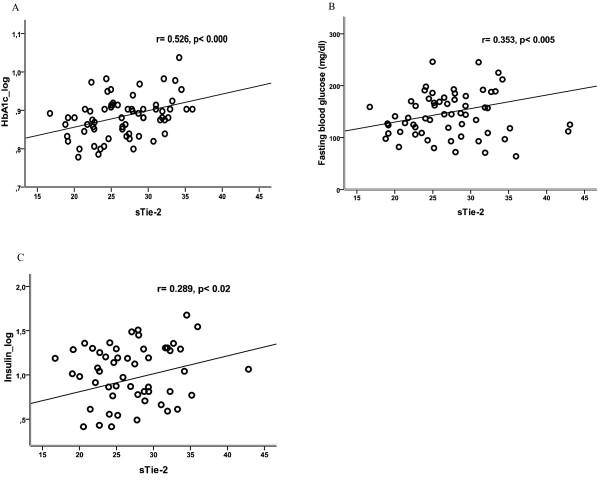
**Associations of sTie-2 with HbA**_1c _**(A), fasting plasma glucose (B), and plasma insulin (C), in type 2 diabetic subjects**.

Studying the relationships of sTie-2 after controlling for age and BMI showed that levels of circulating sTie-2 positively associated with HbA**_1c _**(Figure 2A), fasting plasma glucose (Figure 2B), insulin (Figure 2C), HOMA-IR, serum triglyceride levels and liver function parameters (AST, ALT and GGT, all p < 0.05) (Table 2). Further controlling for levels of serum triglyceride and liver function parameters showed that sTie-2 levels still remained significantly associated with HbA**_1c _**(r = 0.453, p < 0.000). Moreover, linear multiple regression analysis (Table 4), indicated that HbA**_1c_**, insulin levels and HOMA-IR independently associated with sTie-2 levels in type 2 diabetic subjects. No significant associations were observed between levels of sTie-2 and anthropometric data and with parameters of renal function, blood pressure, presence of micro-and macrovascular complications, and type of diabetic therapy. In addition, there were no significant associations between levels of sTie-2 and the plasma levels of testosterone in male as well as plasma levels of estradiol in female participants.

**Table 4:** Multiple regression analysis with levels of circulating sTie-2 as dependent factor in type 2 diabetic participants

Parameters	Standard error	*p*-values
Age	0.111	0.12

*HbA_1c_	15.174	**0.01**

Fasting blood glucose	0.078	0.06

*Insulin	24.553	**0.03**

*HOMA-IR	24.696	**0.03**

Triglyceride	0.007	0.10

*AST	8.244	0.61

ALT	0.089	0.07

*GGT	2.627	0.06

Significant *p-values *are highlighted in bold

(*): data not normally distributed and log_10- _transformed for analysis. HOMA-IR: homeostasis model assessment for insulin resistance, AST: aspartate aminotransferase, ALT: alanin aminotransferase, GGT: gamma-glutamyl transferase.

## Discussion

This is the first study simultaneously investigating circulating levels of Ang-2 and sTie-2 in subjects with established type 2 diabetes mellitus. We found that: (1) Levels of Ang-2 are higher among subjects with than without overt macrovascular complications. (2) Levels of serum GGT predict levels of circulating Ang-2. (3) Levels of sTie-2 are independently associated with HbA**_1c_**, plasma insulin levels and HOMA-IR. (4) There are no significant associations between levels of sTie-2 and the presence of micro-and macrovascular complications including diabetic polyneuropathy. (5) There are no gender differences in levels of Ang-2 and sTie-2 in diabetic subjects.

### Potential effects of Ang-2 in diabetic patients

It is well established that Ang-2 modulates endothelial cell biology and destabilizes blood vessels to facilitate angiogenesis. Ang-2 is a key angiogenic hypoxia-induced growth factor [23]. Increasing evidence suggests that targeting the Ang-2/Tie-2 signaling pathway to inhibit the function of Tie-2 expressing macrophages might extend the effect of vascular-targeting therapies in cancer patients [24]. Also, restoration of Ang-2 to Ang-1 ration may be a novel therapeutic strategy for the treatment of diabetic myocardial ischemic disease [25]. Moreover, in diabetes mellitus, chronic hyperglycemia causes an accelerated formation of advanced glycation end products (AGE) and mitochondrial overproduction of reactive oxygen species (ROS). The resulting toxic and oxidative stress in vascular endothelium promotes micro-and macrovascular complications [26,27]. Amongst multiple pathological changes in gene expression, AGE and ROS lead to the up-regulation of Ang-2 mRNA expression [28], which promotes vascular permeability, destabilization and sprouting [29]. In this study, subjects with diabetic macrovascular complications, in particular those with cardiovascular disease had higher serum levels of Ang-2 than subjects without macrovascular complications. This is supported by a previous *in vitro *study, showing that hyperglycemia causes an increase in Ang-2 leading to increased myocardial apoptosis, increased infarction size and impaired myocardial angiogenesis [25]. Furthermore, in this study, we found that levels of serum GGT independently predict levels of circulating Ang-2 in type 2 diabetic subjects. GGT has been shown to be directly involved in the generation of ROS [30]. Serum levels of GGT were previously reported as markers of oxidative stress [31,32] and have been shown to be associated with hypertension, cardiovascular disease and peripheral vascular disease [33,34]. Therefore, our finding of high levels of serum GGT as an independent predictor for Ang-2 levels in type 2 diabetes mellitus could explain the association of type 2 diabetes and Ang-2 and further supports the hypothesis that therapeutic strategies for reduction of oxidative stress in diabetes mellitus might lower the incidence of micro-and macro-vascular complications [35,36]. In addition, recent data showed that pharmaco-therapeutic interventions using pioglitazone can modulate vascular remodeling biomarkers such as VEGF concentration in ischemic tissue independent of peroxisome proliferator-activated receptor gamma stimulation [37].

### Potential effects of sTie-2 in diabetic patients

On the other hand, Tie-2 appears to be important for angiogenic remodeling and vascular stabilization and has a functional role in pathological angiogenesis in adult tissue [38]. However, the exact role of the soluble form of his receptor in plasma is still not understood. sTie-2 has been shown to inhibit angiogenesis by limiting circulating Ang-1 and Ang-2 from presentation to tissue endothelial Tie-2, with subsequent loss of vessel stabilization signaling [39]. Elevated plasma levels of sTie-2 have been observed in states of inflammation, malignancies [40], type 2 diabetes and coronary heart disease [41]. In this study, there were no direct associations between serum levels of sTie-2 and the presence of diabetic micro-and macrovascular complications, but we found that levels of HbA**_1c_**, insulin, and HOMA-IR are independent predictors for sTie-2 levels in type 2 diabetic subjects. Hyperinsulinemia and insulin resistance contribute to vascular injury and the atherosclerotic process [42] and it is well established that in patients with type 2 diabetes the risk of diabetic vascular complications is strongly associated with the degree of glycaemic control [43]. Indeed, the potential association of the levels of sTie-2 and HbA**_1c _**that has been observed in this study is consistent with the results of previous *in vivo *observations [25]. Hyperglycemia disturbs the Angiopoietin/Tie-2 system toward lowering the tissue Tie-2 expression and increasing circulating plasma sTie-2 hence might contribute to an impaired angiogenesis.

Moreover, recent studies on non-diabetic subjects showed higher plasma levels of Ang-2 in women than in men and that levels Ang-2 and sTie-2 are modulated by estrogen [17,44]. Our data show no significant differences in levels of Ang-2 and sTie-2 between men and women with diabetes, which might be due to the fact that the study included only elderly diabetic men and post-menopausal women (age 65 ± 7 years). Further studies in pre-menopausal women are required to address this issue.

### Limitations of the study

It has to be stressed that this present study is limited by its cross-sectional design. Although the mechanisms mentioned can describe the associations of Ang-2 as well as sTie-2 with type 2 diabetes mellitus, the causal relationship is still unclear. Longitudinal studies on type 2 diabetic subjects could provide a better basis to elucidate the exact impact of sTie-2 in the pathogenesis of diabetes and its complications.

## Conclusion

Our data show that levels of serum GGT, which may serve as a maker for oxidative stress, could predict Ang-2 levels, which further relate to macrovascular disease, while HbA**_1c_**, insulin levels and HOMA-IR might predict levels of circulating plasma sTie-2 in type 2 diabetic subjects. In addition, the study demonstrates that there is no gender specific difference in the levels of Ang-2 and sTie-2 in elderly type 2 diabetic subjects with comparable distributions of diabetic complications. Therefore, circulating Ang-2 could serve as a new marker of oxidative stress and vasculopathy while sTie-2 seems to primarily reflect long-term metabolic control in advanced type 2 diabetes.

## List of abbreviations

AGE: Advanced glycation end product; Ang: Angiopoietin; ALT: Alanin aminotransferase; AST: Asparate aminotransferase; BMI: Body mass index; GFR: Glomerular filtration rate; GGT: Gama glutamyl-transferase; HbA**_1c_**: Glycated hemoglobin; HOMA-IR: Homeostasis model assessment of insulin resistance; ROS: Reactive oxygen species; sTie-2: Soluble Tie-2; Tie-2: Endothelium specific receptor tyrosine kinase; VEGF: Vascular endothelial growth factor.

## Competing interests

The authors declare that they have no competing interests.

## Authors' contributions

SR researched data, wrote manuscript, contributed to discussion. MHR wrote manuscript. AI researched data. KL researched data. LW researched data, reviewed/edited manuscript. AK-W wrote manuscript, contributed to discussion, reviewed/edited manuscript. All authors have read and approved submission of the final manuscript.
